# Coagulation Activation in Children with Sickle Cell Disease Is Associated with Cerebral Small Vessel Vasculopathy

**DOI:** 10.1371/journal.pone.0078801

**Published:** 2013-10-25

**Authors:** Raffaella Colombatti, Emiliano De Bon, Antonella Bertomoro, Alessandra Casonato, Elena Pontara, Elisabetta Omenetto, Graziella Saggiorato, Agostino Steffan, Tamara Damian, Giuseppe Cella, Simone Teso, Renzo Manara, Patrizia Rampazzo, Giorgio Meneghetti, Giuseppe Basso, Maria Teresa Sartori, Laura Sainati

**Affiliations:** 1 Clinic of Pediatric Hematology-Oncology, Department of Pediatrics, Azienda Ospedaliera- Università di Padova, Padova, Italy; 2 Department of Cardiologic, Thoracic and Vascular Sciences, Azienda Ospedaliera- Università di Padova, Padova, Italy; 3 Department of Diagnostic Laboratory and Cellular Therapy, Centro Riferimento Oncologico, Aviano, Italy; 4 Università di Padova, Padova, Italy; 5 Neuroradiology Unit, Azienda Ospedaliera-Università di Padova,Padova, Italy; 6 Neurosonology Unit, Department of Neurological Sciences, Azienda Ospedaliera-Università di Padova,Padova, Italy; Liverpool School of Tropical Medicine, United Kingdom

## Abstract

**Background:**

Thrombotic complications in Sickle Cell Disease (SCD) arise since infancy, but the role of the coagulation system in children has been poorly explored. To determine its role in the development of clinical complications in childhood we measured coagulation and endothelial parameters in children with SCD at steady state.

**Methods:**

Markers of thrombin generation, fibrin dissolution and endothelial activation were evaluated in 38 children with SS-Sβ°, 6 with SC disease and 50 age and blood group matched controls. Coagulation variables were correlated with markers of hemolysis and inflammation, with the presence of cerebral and lung vasculopathy and with the frequency of clinical complications.

**Results:**

SS-Sβ° patients presented higher levels of factor VIII, von Willebrand factor antigen (VWF:Ag) and collagen binding activity, tissue plasminogen activator antigen (t-PA:Ag), D-dimer, p-selectin, prothrombin fragment1+2 (F1+2) and lower ADAMTS-13:activity/VWF:Ag (p<0.05) compared to controls and SC patients. In SS-Sβ° patients coagulation variables correlated positively with markers of inflammation, hemolysis, and negatively with HbF (p<0.05). Patients with cerebral silent infarcts showed significant decrease in t-PA:Ag and ADAMTS-13 Antigen and a tendency toward higher D-dimer, F1+2, TAT compared to patients without them. D-dimer was associated with a six fold increased risk of cerebral silent infarcts. No correlation was found between coagulation activation and large vessel vasculopathy or other clinical events except for decreased t-PA:Ag in patients with tricuspid Rigurgitant Velocity >2.5m/sec.

**Conclusions:**

SS-Sβ° disease is associated with extensive activation of the coagulation system at steady state since young age. ADAMTS-13 and t-PA:Ag are involved in the development of cerebral silent infarcts.

## Introduction

Sickle Cell Disease (SCD) is one of the most common severe monogenic disorders worldwide. Its most frequent variant (Sickle Cell Anemia or homozygous SS disease) is caused by a single amino acid substitution at the sixth residue of the β-globin subunit (β6-Glu →Val) which results in the production of the characteristic sickle hemoglobin. Several double heterozygous forms give also rise to the clinical picture of SCD. The double etherozygous Sβthalassemia° (S mutation coupled with a thalassemia β° mutation) is the most severe form with a clinical picture similar to SS disease, while the double etherozygous SC disease (in which the hemoglobin composition is approximately 50% hemoglobin S and 50% hemoglobin C) displays intermediate severity. Despite being a monogenic disorder, SCD presents with extreme phenotypic variability. Hemolytic anemia, vaso-occlusion and vasculopathy are the hallmarks of SCD pathophysiology, but it’s now clear that multiple actors including leukocytes, platelets, endothelial cells, proinflammatory cytokines, oxidative stress and reduced nitric oxide (NO) availability, and hemostatic activation play a role in disease expression [1-3]. Although thrombotic manifestations and organ damage develop since early infancy, the coagulation system in children with SCD has been poorly explored. Increased thrombin generation and fibrin dissolution are present in children with SCD [4] and, recently, evidence of a procoagulant potential in their plasma has also emerged [5-7]. In adults with SCD, D-dimer and thrombin-antithrombin complexes (TAT) significantly correlate with a history of stroke and retinopathy, respectively [8] and hypercoagulability showed a certain degree of correlation with the development of pulmonary hypertension [9]. Increased von Willebrand factor antigen (VWF:Ag) and high molecular weight multimers have been associated with nocturnal hypoxemia in children with SCD [10]. However, it is still not clear whether the activation of the coagulation system is a bystander phenomenon or a main determinant of clinical complications. Moreover, the association of hypercoagulability with specific clinical manifestations of SCD in childhood has not been reported. 

Since both the physiology of hemostasis and the clinical expression of SCD in pediatric patients differ widely from that in adults [11,12], studies are needed to unravel the possible role of the coagulation system in the development of clinical complication in children with SCD, when the extensive organ damage observed in adults has not developed and the alterations in the endothelium might be reversible. The chronic vasculopathy of SCD is multi-organ [13] and can clinically manifests itself, among others, as cerebral vasculopathy (stroke and silent infarcts, i.e ischemic lesions usually affecting the white matter and the basal ganglia demonstrated by neuroimaging in patients without focal neurological symptoms, generally associated with a decline in neurocognitive function), as lung vasculopathy (impaired lung function and pulmonary hypertension), and as vasoocclusive crisis. While Transcranial Doppler (TCD) or Transcranial Doppler Imaging (TCDi), Magnetic Resonance Imaging (MRI) and Magnetic Resonance Angiography (MRA) allow diagnosis of the cerebral vasculopathy once it is already clinically evident [14,15] and cardiac ultrasound allows suspecting of pulmonary vasculopathy through Tricuspid Regurgitant Velocity (TRV) measurement [16,17], biological markers that could be clinically correlated with organ-specific vasculopathies in the pediatric age are still lacking. Indentifying specific markers of systemic or organ-specific vasculopathy could guide new targeted therapies [18].

We evaluated thrombin generation, fibrin dissolution and markers of endothelial activation in children with SCD at steady state and investigated their role in the development of the most frequent clinical manifestations of SCD in childhood. 

## Methods

### Study Population

Children with SCD above one year of age who consecutively attended for comprehensive care [19] the Clinic of Pediatric Hematology-Oncology of the Azienda Ospedaliera-Università di Padova’s Hospital between January 1^st^ and December 31^st^ 2011 and not on chronic transfusion, were proposed for this cross-sectional study. Patients with SS and Sβthalassemia° (Sβ°) where analyzed separately from patients with SC disease.

In our Center patients are prescribed Amoxacyllin prophylaxis since diagnosis (oral Penicillin is not available in Italy). Children are scheduled to perform a complete check-up every year and to be clinically evaluated at least every 4 months. The complete check-up includes TCD and TCDi starting at age two, echocardiography starting at age three, MRI and MRA of the brain starting at age 5, or when sedation is no longer necessary (unless clinical reasons require them to be performed earlier). Fifty age and blood group-matched controls were recruited among healthy children who accessed the coagulation laboratory for general screening. 

Laboratory analyses, ecochardiography and neuroimaging studies were performed at steady state, defined as a ≥4 weeks from an acute illness and ≥10 weeks post-transfusion. Clinical events were defined according to commonly used criteria [20,21]. Clinical evaluation and laboratory tests were performed on the same day of TCD/TCDi and within one month of an MRI/MRA and cardiac ultrasound. Patients’ data were recorded on the Sickle Cell Access Database in use at our Center. 

### Ethic Statement

The medical ethic committee of our institution Azienda Ospedaliera-Università di Padova approved the study. Written informed consent was obtained from the caregivers on behalf of the children, according to the Declaration of Helsinki.

### Cerebral and lung vasculopathy

TCD and TCDi were performed using a 2 MHz pulsed Doppler ultrasonograph (EME TCD 2000/S) and a ATL HDI 3000/S Echo Doppler system respectively. Patients were not sedated during the examination. We have measured peak systolic blood flow velocities, end diastolic blood flow velocities, time-averaged mean velocity of maximum blood flow (TAMM) and mean blood flow velocities, at the level of the Internal Carotid Artery and of the Middle Cerebral Artery on both sides of the brain. We used the Stroke Prevention Trial in Sickle Cell Anemia Study (STOP) criteria [14] to assign stroke risk as low (TAMM <170 cm/sec), conditional (TAMM 170 - 199 cm/sec), or high (TAMM ≥ 200 cm/sec). The same criteria were used for evaluation of TCDi.

MRI and MRA studies were performed as previously described [22]. Cardiac ultrasound and TRV measurement were performed as previously described [23].

### Laboratory data

Blood samples were drawn between 8:00 and 10:00 a.m., following an overnight fast and without venous stasis. Nine ml of whole blood were anticoagulated with 1 ml of trisodium citrate (0.13 M); after centrifugation at 2671 xg for 15 minutes, plasma aliquots were stored at -40°C, and tested within one month. 

PT, PTT were assayed by standard methods. Commercially available enzyme-linked immunosorbent assays (ELISA) were used to measure: Plasminogen activator inhibitor-1 antigen (PAI-1:Ag; ZYMUTEST PAI-1 Antigen, Hyphen BioMed, Neuville-sur-Oise, France), tissue plasminogen activator antigen (t-PA:Ag; TriniLYZE t-PA antigen, Trinity Biotech, New York, USA), D-dimer (ZYMUTEST DDimer, Hyphen BioMed), P-selectin (Human sP-selectin, R&D Systems, Minneapolis, MN, USA), TAT (Enzygnost TAT micro; Siemens Healthcare Diagnostics, Marburg, Germany), F1+2 (Enzygnost® F1 + 2, Siemens Healthcare Diagnostics, Marburg, Germany), ADAMTS13 antigen (ADAMTS13:Ag) and activity (ADAMTS13:act) (TECHNOZYM® ADAMTS13 antigen, Technoclone, Vienna, Austria). Nitric Oxide (NO) was determined using a quantitative colorimetric commercial assay (Nitrate/Nitrite colorimetric Assay Kit, Cayman Chemical Company, Ann Arbor, Michigan, USA), Factor VIII activity (FVIII) was measured using a one-stage clotting method (BCS-XP 4 Siemens Healthcare Diagnostics, Milan, Italy). VWF:Ag was determined by a home-made ELISA method, using a horseradish peroxidase (HRP)-conjugated anti-VWF antibody (Dako, The Netherlands) while its collagen binding activity (VWF:CB) was assessed by ELISA using type I and type III collagen diluted in acetic acid solution (95% and 5%, respectively). After overnight coating with collagen, microtiter plates were incubated with plasma VWF for 1 hour at room temperature; bound VWF was evaluated with HRP-conjugated anti-VWF antibody.

Markers of hemolysis (hemoglobin, reticulocytes, aptoglobin, total and indirect bilirubin, lactate dehydrogenase, aspartate transaminase) and inflammation (white blood cells, neutrophils and C reactive protein) were measured by standard automated laboratory procedures.

### Statistical Analyses

Statistical calculations were made by SAS (release 9.1.3; SAS Institute, Cary, North Carolina) and SPSS softwares (release 18.0.0; SPSS inc, Chicago, Illinois).

Continuous variables were summarized as means and standard deviations and compared using the two-sided Student's t-test. Categorical variables were summarized as proportions and compared using the Chi-square test or the Fisher's exact test (whichever was appropriate). Correlations between variables were evaluated by the Pearson or Spearman coefficient. Relative risk (RR) was used to measure the strength of the association between binary variables. P-values <0.05 were considered statistically significant. 

## Results

### Population characteristics and clinical events

Coagulation analysis was performed on thirty-eight SS-Sβ° and six SC patients. SS-Sβ° patients were 20 Males and 18 Females, mean age 6.49 years (range 1.48-15.11 years); 35 were SS and 3 Sβ°, 36/38 (94.7%) patients were Africans while 2/38 were Albanians. Five patients were on Hydroxiurea (HU) treatment for recurrent acute chest syndrome (n°4) or persistent severe anemia (n°1). None of the 38 patients presented with stroke, one patient had seizures and is currently undertaking antiepileptic treatment. Neurological evaluation was normal in all patients. Twenty-four out of 38 patients (63%) had experienced at least one acute chest syndrome, with 17/38 experiencing between 2 and 7 episodes. Thirty-one out of 38 children (81.5%) had presented at least one vaso-occlusive crisis requiring hospitalization or Emergency Room visit and 19/38 had experienced between 2 and 8 vaso-occlusive crisis. Ten out of 38 patients presented hemolytic crisis, 6/38 aplastic crisis and 2/38 splenic sequestration. Thirty-six out of 38 children (94.7%) had been admitted at least once (range 0-24). No episodes of priapism, avascular necrosis or leg ulcers were observed in our cohort. 

SC patients, 3 Males and 3 Females, mean age 11.2 years (range: 4-18.2), were all Africans and presented less acute chest syndromes (17%), vaso-occlusive crisis (50%), hemolytic crisis (17%) and no aplastic crisis or splenic sequestration.

All patients were receiving amoxicillin prophylaxis and folic acid; all had completed the regular vaccination schedule, including pneumococcus and meningococcus vaccination.

### Hematological and Coagulation analyses

Hematological and coagulation profiles are shown in Table 1 and 2, respectively. As shown in Table 2, SS-Sβ° patients showed significantly increased markers of endothelial activation, thrombin generation and fibrinolyis compared to controls. Specifically, SS-Sβ° patients presented increased VWF:Ag and VWF:CB, but the VWF:CB/VWF:Ag ratio was found significantly decreased, suggesting a less pronounced representation of large VWF multimers. These findings were also maintained when the data were analyzed respect to ABO blood groups, according to the lower VWF levels observed in normal subjects with blood group O [24,25]. ADAMTS activity and ADAMTS-13:Act/VWF:Ag were also significantly decreased in SS-Sβ° compared to controls. P-selectin was increased and NO decreased compared to controls, further supporting the presence of endothelial dysfunction in children affected by SS-Sβ°. Less pronounced hemostatic alterations were observed in 6 SC patients, showing a mild perturbation of endothelial cells, abnormal fibrinolysis but no signs of increased thrombin generation (Table 2).

**Table 1:** Hematological Values of 38 children with SS-Sβ° and 6 with SC Sickle Cell Disease.

	**SS-Sβ°** (Mean ±SD)	**SC** (Mean ±SD)	**p-value**
Age (years)	6.49 ±3.48	10.09 ± 6.1	ns
Systolic Blood Pressure (mmHg)	105 ±10.78	105.67 ± 10.48	ns
Dyastolic Blood Pressure (mmHg)	62.78 ±10.49	68.83 ± 4.36	ns
SpO_2_ (%)	98.32 ±1.76	99.67 ± 0.82	0.009
Haptoglobin (g/L)	.1857 ±.22	nd	nd
Indirect Bilirubin (μg/L)	29.129 ±15.53	21.38±12.67	ns
Total Bilirubin (μg/L)	35.537 ±17.19	27.15±16.13	ns
Direct Bilirubin (μg/L)	6.542 ±3.19	5.77±4.03	ns
**Lactate Dehydrogenase (U/L)**	1158.47 ±321.14	601.83±136.87	0.01
**Reticulocytes (10^9^/L)**	242428.95 ±75236.64	134133.33±23615.05	0.01
**Hemoglobin (g/dL)**	8.761 ±1.27	11.38±0.98	0.01
**Hb F (%)**	13.213 ±8.03	3.78±2.05	0.01
**Hb S (%)**	76.045 ±9.48	45.2±5.49	0.01
Mean Corpuscolar Volume (fL)	82.21 ±10.43	78.43±9.17	ns
**White Blood Cells** (10^9^/L)	13150.00 ±4180.44	7953.33±3131.3	0.01
**Neutrophils (10^9^/L)**	6139.19 ±2615.93	3366.67±1717.75	0.01
**Platelets (10^9^/L)**	387.42 ±141.68	301.16±472.79	0.01
Ferritin (μg/L)	157.42 ±103.38	127.8±89.25	ns
Creatinine (μg/L)	36.32 ±9.66	45.83±10.59	ns
Urea (mmol/L)	3.10 ±.93	9.91± 5.81	ns

**Table 2:** Markers of endothelial activation, thrombin generation and fibrinolysis for SS/Sβ° patients, SC patients and Controls.

	SS/Sβ°	SC	Controls	p-value		
	N°38	N°6	N°50	SS vs Controls	SC vs Controls	SS/Sβ° vs SC
	Mean ± SD	Mean ± SD	Mean ± SD			
*Markers of endothelial activation*						
FVIII (60-160%)	184.96 ±62.80	119.22 ±20.99	100.09 ±31.61	0.001	0.050	0.003
VWF:Ag (60-160%)	150.05 ±49.75	100.63 ±27.91	92.39 ±24.94	0.001	0.368	0.013
VWF:CB (65-150%)	135.38 ±40.31	107.36 ±32.93	94.15 ±27.58	0.001	0.272	0.124
VWF:CB/VWF:Ag	0.9186 ±.11	1.06 ±.7	0.9960 ±.09	0.001	0.116	0.005
ADAMTS13 Ag (0.6-1.6 μg/ml )	1.37 ±.49	1.46 ±.31	1.10 ±.40	0.002	0.037	0.653
ADAMTS13 Act (40-130%)	79.42 ±12.82	78.94 ±5.17	85.00 ±6.2	0.013	0.035	0.801
ADAMTS13 act/VWF:Ag	0.52 ±.11	0.78 ±.24	0.92 ±.21	0.001	0.452	0.003
P-selectin (18-40 ng/ml)	47.76 ±18.31	29.70 ±7.78	29.00 ±4.68	0.001	0.850	0.010
NO (12.2–69.4 μmol/L)	7.18 ±3.63	8.7 ±4.04	40.8 ±5.10	0.001	0.001	0.336
*Markers of thrombin generation and fibrinolysis*						
F1+2 (69-229 pmol/l)	254.26 ±292.01	118.50 ±36.50	149.00 ±27.12	0.037	0.096	0.037
TAT (1.0-4.1 ng/ml)	12.34 ±21.28	5.14 ±2.57	2.55 ±1.61	0.009	0.057	0.359
D-dimer (<400 ng/ml)	1906.39 ±1515.70	366.48 ±151.26	250 ±110.11	0.001	0.118	0.001
PAI-1:Ag (1-25 ng/ml)	8.73 ±7.02	6.85 ±3.73	13.00 ±2.65	0.001	0.010	0.833
t-PA:Ag (2-12 ng/ml)	6.91 ±3.51	4.52 ±3.51	7.00 ±2.84	0.880	0.022	0.062

Factor VIII (FVIII), von Willebrand factor antigen (VWF:Ag) and collagen binding activity (VWF:CB), ADAMTS13 antigen (ADAMTS13:Ag) and activity (ADAMTS13:act), Nitric Oxide (NO), Prothrombin Fragment 1+2 (F1+2), Thrombin Antithrombin Complexes (TAT), Plasminogen activator inhibitor-1 antigen (PAI-1:Ag), tissue plasminogen activator antigen (t-PA:Ag).

### Cerebral and lung vasculopathy

TCD and TCDi were performed on 35/38 SS-Sβ° patients older than two years of age. Three patients displayed abnormal values at TCD and were successively placed on a chronic transfusion program, according to the guidelines for primary stroke prevention [14]. The remaining were normal. TCDi displayed similar results except allowing a better definition of the Internal Carotid Artery. Thirty patients were above 5 years of age and performed MRI/MRA. Nine (30%), presented Cerebral Silent Infarcts on MRI: 5 with a total lesion volume < 500 mm^3^, 4 with a total lesion volume >500 mm^3^. None of the patients showed vascular occlusion on MRA, and 23/30 (76.6%) displayed different grades of stenosis: 5 presented a mild stenosis (grade 1) and 18 a severe stenosis (grade 2).

Cardiac Ultrasound was performed on 31 patients. Cardiac anatomy was normal in all. Seven out of 31 (29.1%) had TRV ≥2.5 m/sec, while the remaining was normal. Mean TRV of the entire population was 2.29 (SD 0.24, range: 1.77-2.75).

All SC patients performed evaluation of cerebral and lung vasculopathy: TCD/TCDi and MRI/MRA were normal and TRV was <2.5 cm/sec in all of them.

### Correlations between the coagulation system, clinical manifestations and vasculopathy

The significant correlations between hematological and coagulation variables for patients with SS-Sβ° are shown in Table 3. Markers of inflammation and hemolysis significantly correlated with the majority of the coagulation parameters. Negative correlation was demonstrated only with hemoglobin F. In SC patients no correlation was seen between markers of inflammation and coagulation while limited correlations were demonstrated with haemolytic markers: LDH correlated positively with VWF:Ag and VWF:CB (0.81, p 0.04; 0.86, p 0.02, respectively) and negatively with NO (-0.95; p 0.42); PAI:Ag correlated with Reticulocytes (0.93, p 0.007).

**Table 3:** Significant correlations between hematologic and coagulation parameters in patients with SS-Sβ°.

	WBC	N	PLT	HbF	Retic	LDH
FVIII	ns	ns	ns	-0.37; 0.02	ns	ns
VWF:Ag	ns	ns	ns	-0.32; 0.04	ns	ns
VWF:CB	ns	ns	ns	ns	ns	0.36; 0.02
ADAMTS-13:Ag	ns	ns	0.47; 0.00	ns	ns	ns
P-selectin	0.46; 0.00	0.40; 0.01	0.52; 0.00	-0.41;0.01	ns	0.38; 0.02
F1+2	0.40; 0.01	ns	ns	ns	0.51; 0.00	0.33; 0.04
TAT	0.39; 0.01	ns	ns	ns	0.38; 0.02	0.37; 0.02
D-dimer	0.55; 0.00	0.43; 0.01	ns	-0.39; 0.02	0.38; 0.02	0.42; 0.01
PAI:Ag	0.44; 0.00	ns	0.36; 0.02	ns	0.33; 0.04	0.35; 0.03
t-PA:Ag	ns	ns	ns	ns	0.34; 0.03	ns

In each column, the first number represents the Spearman’s or Pearson’s correlation coefficient and the second number represents significance (p-value<0.05).White Blood Cells (WBC), Neutrophils (N), Platelets (PLT), Reticolocytes (Retic), Lactate Dehydrogenase (LDH), Factor VIII (FVIII), von Willebrand factor antigen (VWF:Ag) and collagen binding activity (VWF:CB), ADAMTS13 antigen (ADAMTS13:Ag), Prothrombin Fragment 1+2 (F1+2), Thrombin Antithrombin Complexes (TAT), Plasminogen activator inhibitor-1 antigen (PAI-1:Ag), tissue plasminogen activator antigen (t-PA:Ag); not significant (ns)

In SS-Sβ° patients a significant negative correlation was demonstrated between lesion dimension of cerebral silent infarcts on MRI and both t-PA value (-0.42, p 0.019) and ADAMTS-13 Ag (-0.39, p 0.03). Increased D-dimer was associated with a significant Relative Risk (RR 6.0, CI 95% 2.45 - 14.68, p <0.05) to develop cerebral silent infarcts. In fact, differences were demonstrated in the level of some coagulation factors between patients with (n.9) and without (n.21) cerebral silent infarcts on MRI (Table 4). Patients with cerebral silent infarcts presented significantly lower mean t-PA:Ag and ADAMTS13:Ag levels and higher D-dimer, F1+2 and TAT levels, although these latter did not reach statistical significance. No differences were observed in mean Oxygen saturation, Blood Pressure, gender, mean hematologic values (haemoglobin, reticulocytes, LDH, indirect bilirubin), nor clinical manifestations (rate of acute chest syndrome, vaso-occlusive, hemolytic or aplastic crisis) between patients with and without cerebral silent infarcts.

**Table 4:** Comparison of mean haematological and coagulation variables ± SD between SS- Sβ° patients with Silent Infarcts and without Silent Infarcts.

**Variable**	**Silent Infarcts**	**No Silent Infarcts**	**p-value**
	**N°9**	**N°21**	
Hemoglobin (g/dL)	8.76 ±1.94	8.68 ±1.09	0.65
Hemoglobin F%	9.07 ± 4.53	12.78 ± 8.80	0.42
Hemoglobin S%	79.18 ± 10.24	75.93 ± 10.13	0.35
Reticulocytes (10^9^/L)	202100.00 ± 78886.26	259476.19 ± 67669.83	0.67
LDH	1233.00 ± 346.12	1190.62 ± 327.31	0.78
PT (%)	69.88 ± 4.64	71.65 ± 6.99	0.63
PTT (s)	25.88 ± 1.55	24.94 ± 1.71	0.28
FVIII:C (%)	167.43 ± 61.50	195.54 ± 67.17	0.16
VWF:Ag (%)	134.25 ±47.55	158.53 ±51.15	0.24
VWF:CB (%)	122.03 ± 38.16	141.02 ± 37.74	0.19
PAI:Ag (ng/ml)	7.77 ± 7.24	9.37 ± 5.88	0.31
***t-PA:Ag (ng/ml)***	***4.53 ± 1.47***	***7.67 ± 3.9***	***0.03***
D-dimer (ng/ml)	2879.01 ± 2813.58	1569.51 ± 829.59	0.78
P-selectin (ng/ml)	52.56 ± 27.71	48.84 ±14.99	0.88
F1+2 (pmol/L)	319.63 ± 564.85	231.86 ±112.40	0.08
TAT (ng/ml)	17.17 ± 37.23	10.46 ±13.73	0.50
***ADAMTS-13 Ag (μg/ml)***	***1.15 ± 0.38***	***1.50 ± 0.54***	***0.05***
ADAMTS-13 act (%)	76.12 ±12.12	83.63 ± 13.01	0.25

Fctor VIII (FVIII), von Willebrand factor antigen (VWF:Ag) and collagen binding activity (VWF:CB), ADAMTS13 antigen (ADAMTS13:Ag) and activity (ADAMTS13:act), Nitric Oxide (NO), Prothrombin Fragment 1+2 (F1+2), Thrombin Antithrombin Complexes (TAT), Plasminogen activator inhibitor-1 antigen (PAI-1:Ag), tissue plasminogen activator antigen (t-PA:Ag).

No correlation was seen between coagulation parameters and cerebral large vessel vasculopathy defined either by TCD, TCDi or by MRA (all p >0.05) on bivariate analysis. No correlation was also shown between coagulation parameters and TRV values, except for a significant negative correlation with t-PA:Ag (-0.36, p 0.04). No correlation was demonstrated between coagulation parameters and the rate of acute chest syndrome, vaso-occlusive crisis, splenic sequestration, hemolytic or aplastic crisis (all p >0.05). 

The correlation analysis between coagulation and vasculpathy was not performed for SC patients due to the lack of abnormal data in these patients.

When the 5 patients on HU treatment were removed from the analysis, the results did not substantially change.

## Discussion

In this study we correlate for the first time the activation of the coagulation system with specific clinical manifestations in young children with SS-Sβ° and suggest its possible role in the development of cerebral silent infarcts but not in cerebral large vessel vasculopathy or other organ-specific vasculopathies. 

We confirm the significant increase of thrombin generation and fibrin dissolution already documented in older patients [8,9]. More importantly, we demonstrate in these children a stronger endothelial activation (increased VWF:Ag, VWF: CB, P-Selectin, decreased ADAMTS13 act/VWF:Ag) compared to children with SC disease and to blood group matched controls. Increased Factor VIII during steady state has already been reported in adults in Nigeria and Jamaica [26,27] and in a small group of 12 children in steady state [28]. Similarly, VWF enhanced the adhesiveness of sickled erythrocytres to the endothelium in vitro [29,30], suggesting its involvement in the vasoocclusive pathophysiology of SCD, although the evidence of a role in vivo in contributing to specific clinical complications, whether in the macro or the microcirculation, is still limited (10) and needs further investigation. As previously reported in adults [31], no severe ADAMTS13 deficiency was reported in our group, nevertheless children with SS-Sβ° presented significantly lower ADAMTS13 activity and ADAMTS13 act/VWF:Ag. A role for ADAMTS13 in SCD has been suspected from studies in vitro [32,33], but not yet confirmed in vivo. In our group, ADAMTS13:Ag was negatively correlated with the presence of cerebral silent infarcts and with infarct lesion dimension. In children with cerebral silent infarcts, ADAMTS13 Ag and t-PA:Ag were significantly lower suggesting endothelial dysfunction, while several other coagulation variables (D-dimer, P-selectin, TAT, F1+2) tended to be higher suggesting a trend to enhanced clotting activation. A possible relative insufficiency of ADAMTS13 could be present in children who develop silent infarcts: in a situation of chronic endothelial activation the activity of ADAMTS13 may not always suffice to prevent an abnormal increase of VWF:Ag in the already hypercoagulable blood and therefore lead to increased infarcts in the cerebral microvasculature. Cerebral vasculopathy is indeed a complex manifestation of SCD, probably with a multifactor origin, and its risk factors are still incompletely understood. Our data suggest that activation of the coagulation system and endothelial dysfunction could be associated with the presence of silent infarcts –believed to be a small vessel disease- and not with the presence of stenosis on brain MRA or abnormal values on TCD/TCDi–expression of large vessel vasculopathy. Liesner R and colleagues had failed to demonstrate a correlation between TCD and increased thrombin generation or reduction of natural anticoagulants but did not display MRI/MRA data [34]. No clinical or haematological parameters correlated with the development of cerebral silent infarcts in our group, but the relatively low age and the limited number of patients might have prevented the emergence of such correlations as other larger cohorts have instead shown [15,35]. Alternatively, we could also hypothesize that, at similar hematologic (i.e.hemolytic rate) and clinical conditions (SatO_2_, clinical events, gender), coagulation activation could imply adjunctive risk factors for the development of certain clinical manifestations, like cerebral silent infarcts. Indeed, significant qualitative and quantitative differences exist between microvascular responses to inflammation in the brain compared to other organs [18,36]. Endothelial cell phenotypes vary between different organs and it has been suggested that several adhesion receptors (i.e VICAM1 variant) present on the endothelium of/sub-endothelium matrix of the human brain microvasculature might produce abnormal vascular adhesion in cerebral small vessels but not in large ones [37]. Moreover, the slow blood flow due to the lower shear stress in the cerebral microvasculature increases the possibility of the rigid sickle red cells to adhere to the highly adhesive endothelium, while hypoxia and inflammation further contribute to this phenomenon enhancing the transcription of genes involved in vasomotor tone regulation, cell proliferation and coagulation [38]. The pro-thrombotic state and the reduced ADAMTS13, which decreases platelet adhesion and aggregation and down-regulates thrombus formation and inflammation, predispose the microvasculature to ischemic events [39,40]. Interestingly, among all the coagulation and endothelial markers evaluated in our study, only decreased t-PA:Ag was associated with elevated TRV, an ultrasound measure of possible risk of pulmonary hypertension and biomarker of disease severity and systemic vasculopathy in SCD [41]. Patients with SCD at steady state display less releasable t-PA:Ag [42]; it is likely that a common mechanism of reduced t-PA:Ag production by a dysfunctional endothelium or a higher t-PA:Ag consumption might exist in the brain and the lungs. 

Neither the incidence rates of acute chest syndrome, vaso-occlusive crisis, splenic sequestration or haemolytic/aplastic crisis, measures of SCD morbidity in childhood, were associated with markers of coagulation and endothelial activation in our pediatric population; thus, it is likely that these markers do not predict acute events in children. 

The negative correlation of HbF with markers of coagulation (Factor VIII, D-dimer), platelet and endothelial activation (P-selectin, VWF:Ag), supports the hypothesis that HbF and HU could reduce the hypercoagulable state also in pediatric patients, as demonstrated in adults [43]. The small number on children on HU treatment in our cohort limits the possibility to draw definitive conclusions, but it is likely that in the near future, more children will receive HU treatment, due to the results of the BABYHUG study [44]. A wider population of children on HU might allow a better definition of the pathogenetic role of elevated HbS and the impact of HU treatment on the activation of the hemostatic system.

Hemolysis plays a crucial role in the development of hypercoagulability and endothelial dysfunction in children with SCD [1,6,8]. In children with SS-Sβ°, markers of thrombin generation and fibrinolysis were all associated with signs of hemolysis and inflammation as well as markers of endothelial activation, demonstrating a broad involvement of the coagulation system in these processes. 

In our series, coagulation pattern in SC children was found to be similar to the one of the control group, partially explaining the different clinical expression of the disease between SS-Sβ° and SC patients, especially regarding neurocerebral abnormalities. Studies in adults with SC yielded, so far, contrasting results [8,45].

In conclusion, our study demonstrates that SS-Sβ° is associated with comprehensive activation of the coagulation system at steady state since early age and that hypercoagulability plays a role in the development of vascular occlusions in certain organs. Our data strongly suggest evaluating markers of endothelial activation and thrombin generation since young age in a large prospective study to verify their clinical implications. In fact, a more precociuous neuroradiological evaluation together with early-onset longitudinal follow-up of both neuroimaging and coagulation status could give better insight into the role of coagulation activation and endothelial dysfunction in the development of systemic and organ-specific vasculopathy in children with SCD.
